# Bridging experiments and defects’ mechanics: a data-driven toolbox for configurational force analysis

**DOI:** 10.1007/s00366-025-02262-5

**Published:** 2026-01-14

**Authors:** Abdalrhaman Koko, Alya Abdelnour, Thorsten H. Becker, T. James Marrow

**Affiliations:** 1 National Physical Laboratory, Hampton Road, Teddington, TW11 0LW UK; 2 Department of Materials, University of Oxford, Oxford, OX1 3PH UK; 3 Department of Mechanical Engineering, University of Khartoum, Khartoum, Sudan; 4 Centre for Materials Engineering, Department of Mechanical Engineering, University of Cape Town, Cape Town, South Africa

**Keywords:** Configurational forces, Stress intensity factors, Mixed-mode fracture, HR-EBSD, Digital image correlation, Computational toolbox; material testing 2.0

## Abstract

Understanding the mechanical behaviour of defective materials is key to predicting failure and enhancing performance. Traditional fracture mechanics often requires assumptions about geometry and loading that are unavailable in experimental systems. We present a MATLAB-based computational toolbox that extracts configurational forces and mixed-mode SIFs directly from experimentally measured displacement or deformation gradient fields, like digital image/volume correlation and high (angular) resolution electron backscatter diffraction. The toolbox implements path-independent energy integrals, including the *J*- and *M*-integrals, and introduces a novel mode decomposition formulation that isolates mode I–III SIFs contributions without predefined specimen geometries, applied loads, or boundary conditions. Applications to microcracks, dislocations, and fatigue cracks demonstrate its robust, geometry-independent characterisation, which can enable data-driven analysis of defect behaviour in anisotropic and complex materials. The framework is material-agnostic in principle and operates directly on experimental fields; however, its current implementation assumes small-strain kinematics, making it most applicable to linear and anisotropic elastic and elastoplastic materials such as metals and ceramics.

**Supplementary Information:**

The online version contains supplementary material available at 10.1007/s00366-025-02262-5.

## Introduction

When a force ($\:\overrightarrow{F}$) acts on a particle, causing it to move along a trajectory; the work ($\:W$) done by the force is given by the dot product of the force and the displacement vector ($\:\varDelta\:\overrightarrow{r}$),

1 $$\:W=\overrightarrow{F}.\varDelta\:\overrightarrow{r}$$

For more complex trajectories where the force varies, the work is calculated by integration along the particle’s path ($\:{\Gamma\:}$), resulting in the integral form

2 $$\:W={\int\:}_{{\Gamma\:}}^{\:}\overrightarrow{F}.d\overrightarrow{r}$$

Thus, the work for a vector force field is calculated using a line integral. Additionally, the direction of the vector ($\:\overrightarrow{T}$) that is tangential to the trajectory over a small interval is represented by the arc element $\:ds$, so [1]

3 $$\:W={\int\:}_{{\Gamma\:}}^{\:}\overrightarrow{F}.\overrightarrow{T}ds$$

If the vector field is a gradient field ($\:\nabla\:\overrightarrow{F}$), it can be derived from a scalar potential function. In this case, the line integral between two points depends only on the endpoints and is independent of the path taken — the field is said to be conservative. For any closed path enclosing no source or sink, the line integral evaluates to zero. The scalar potential (or potential energy) associated with such a field describes the energy stored by the force field. This concept underpins the conservation of energy and momentum, as captured by Noether’s theorem [2, 3], which states that path-independent integrals correspond to conserved physical quantities in systems with continuous symmetries. In solid mechanics, this principle forms the foundation for configurational force integrals used to describe defect mechanics.

In solid mechanics, surface traction in elastic bodies is described in terms of a vector field with respect to the surface’s initial position. Since the body force density can be considered fixed in elastic bodies, the potential energy is a function of the displacement field [4, 5]. When evaluated over a surface that encloses a stress-concentrating defect, these conservation integrals represent a configurational force on the defect, which may be, for example, an inclusion [6] or dislocation [7, 8]. These integrals are direct consequences of momentum balance. The significance of these integrals in engineering and materials mechanics emerged as researchers sought to quantify the energy dissipation associated with material deformation, especially in the presence of defects. This force, defined as the negative gradient of the material’s total energy with respect to the defect’s position, diverges from traditional Newtonian mechanics by originating not from the material’s physical position in space but rather from the defect’s position within the structure [6, 7]. The configurational force was further elaborated through the development of the Peach-Koehler force [8], which describes the interaction between dislocations and stress fields generated by defects in the elastic medium. This theory was later refined via the divergence of Eshelby’s stress tensor [9, 10], firmly establishing the role of these forces in classical dislocation theory [11].

A major milestone in the field of fracture mechanics came with Rice’s formulation of the *J*-integral [12] as a path-independent integral to quantify the strain energy release rate during crack propagation. The *J*-integral allows for precise evaluations of strain energy dissipation in materials undergoing fracture, including anisotropic, linear, and nonlinear materials [4, 13]. The *J*-integral is central to modern fracture mechanics. It is easy to implement in finite element software, and has been coupled with residual stress, internal tractions, and thermal and electrochemical processes [14], and the analytical solutions for stress intensity factors used in standardised fracture toughness tests are either approximated or derived from the *J*-integral [15]. Further expansion of energy conservation principles led to the introduction of invariant integrals such as *M*-, and *L*-integrals [16, 17], generalising the *J*-integral to account for energy dissipation modes. These invariant integrals have been used to characterise the energy available for defect propagation or transformation [18–20], as they provide a descriptor of the deformation field at stress-concentrating defects, such as complex crack growth scenarios [21–24], dislocations [25, 26], inclusions [27], and slip bands [28].

Stress concentrations influence material failure, making understanding and quantifying how they affect the failure mechanisms is essential. Researchers, such as Lazar [29–31], extended the *J*-, *M*-, and *L*-integrals into gradient elasticity theory, applying them to inhomogeneous, incompatible, and anisotropic materials. These extensions are particularly relevant to materials containing dislocations and disclinations, since classical elasticity theory fails to describe their interactions at the micro-scale.

However, the invariant integrals do not distinguish between mechanical loading conditions (i.e., tensile and compressive mode I, in-plane shear mode II, and out-of-plane shear mode III) at the defects, which is crucial since it is well established in fracture mechanics that these conditions can affect both the defect growth rate and direction [32]. Traditionally, the mode I–III stress intensity factors (SIFs) have been obtained using analytical solutions [33, 34] or finite element methods (FEM), relying on known or idealised loads, boundary conditions, and specimen geometries [35, 36]. However, real-world conditions introduce uncertainties that undermine these approaches [37–39], such as residual stresses [40, 41], frictional effects [42], misalignment [43, 44], and the lack of knowledge of the boundary conditions. This complicates stress field characterisation, especially at the micro-scale where deformation is microstructurally informed. To address these issues, direct full-field measurement techniques, like high (angular) resolution electron backscatter diffraction (HR-EBSD) [45, 46] and digital image correlation (DIC) [47, 48], have gained prominence, offering a more accurate and true assessment of the local stresses that drive the failure and deformation mechanisms.

We present a freely available MATLAB-based toolbox to calculate configurational forces and mixed-mode stress intensity factors from displacement and displacement gradient fields. This toolbox enables precise extraction of defect field descriptors, such as the fracture parameters for cracks in isotropic/anisotropic elastic and elastoplastic materials. By utilising only local field data, the toolbox accurately computes mixed-mode SIFs, without the need for predefined specimen geometries or knowledge of the applied loads. Its ability to handle complex geometries and non-standard defect shapes makes it highly adaptable for real-world applications.

## Theoretical frame and computational implementation

### Numerical evaluation of the *J*-Integral

James Rice [12] formulated a path-independent contour/line integral to capture the configurational forces acting on cracks. Earlier investigations by Sanders [49] and Cherepanov [50] are closely related to Rice’s work. Initially, the James-integral or the *J*-integral^1^ was defined as the strain energy release rate or work/energy per surface area for cracks subjected to monotonic loading in linear-elastic, plastic-elastic, or plastic materials. This is described in Eq. (4) for an arbitrary contour ($\:{\Gamma\:}$) integral around a crack tip, where

${\sigma}_{ij}$ and $\:{\varepsilon}_{ij}$ are the stress and strain tensors, respectively, $\:W$ is the strain energy density, $\:{u}_{i}$ is the displacement vector components, $\:{n}_{j}$ is the components of the unit vector normal to $\:\varGamma\:$, $\:ds$ is the length increment along the $\:\varGamma\:$ and $\:\delta\:$ is the Kronecker delta, which is 1 if =1 and 0 otherwise.

4 $$\begin{aligned} {J_k} & =\mathop \smallint \limits_{\Gamma } \left( {~W{\delta _{kj}} - ~{\sigma _{ij}}{u_{i,k}}} \right){n_j}~d\Gamma ,~~ \\ W & =\mathop \smallint \limits_{0}^{{{\varepsilon _{ij}}}} {\sigma _{ij}}~d{\varepsilon _{ij}}, \:\:k =1,2, ~~\\{\delta _{kj}}=\left\{ {\begin{array}{*{20}{c}} 1&{if~k=j} \\ 0&{if~k \ne j} \end{array}} \right.~ \\ \end{aligned} $$

Hutchinson [51] and Rice and Rosengren [52] were the first to notice the importance of the *J*-integral as a criterion for crack growth for linear elastic or elastoplastic (Ramberg-Osgood power hardening) materials. While the original *J*-integral formulation was derived for monotonic loading in nonlinear elastic materials, its application to elastoplastic systems requires careful extension. In particular, the presence of unloading, crack face tractions, and internal residual stresses renders the energy dissipation history-dependent. To address this, various generalisations of the *J*-integral have been proposed, including domain integral formulations, via the finite element method [53–55], that account for plasticity effects and allow for numerical implementation using stress and strain energy fields, including surface traction [56], loading and internal tractions [57], thermal [58, 59] and electrochemical [60], residual stresses [59] and boundary interactions [61–63]. Subsequently, it was expanded to describe cracks in dynamic loading [64], dissimilar materials [65], and also non-crack defects like dislocations [30, 66] and misfitting inclusions [67] as an equivalent descriptor of forces on the defects or their potential energy release rate^2^ [68, 69].

**Fig. 1:**
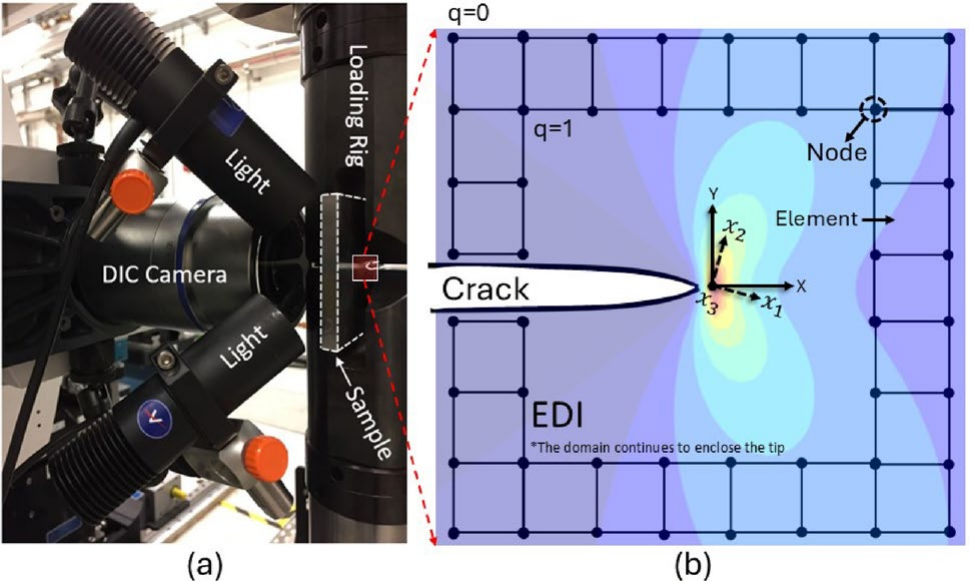
**a** Experimental setup for in-plane displacement field measurement using digital image correlation on a compact tension specimen with a surface speckle. **b** Representation of the strain field ($\:{\varepsilon}_{22}$) overlaid with the crack path and the equivalent domain integration (EDI) scheme. The domain incrementally expands outward from the crack tip, enabling the numerical evaluation of the $\:{J}_{k}$-integral and mode-decomposed SIFs. Each domain contour corresponds to a discrete area increment ($\:dA$) mapped to a regularised measurement grid. The direction of crack extension or virtual crack extension (VCE) vector is assumed along the $\:{x}_{1}$-axis

While the traditional contour (line) integral relies on an idealised path around the crack tip, domain integration – implemented as the equivalent domain integral (EDI) method [54, 70, 71] – distributes the computation over a larger region, which reduces the sensitivity to both local mesh refinement and numerical errors [70–72], while capturing contributions from plastic dissipation and internal tractions more effectively than a traditional path integral. In this method, an integral along a contour is transformed into an area or volume domain integral containing a weight function that undergoes a smooth linear spatial variation across the domain ($\:\frac{d{q}_{k}}{d{x}_{1}}$ in Eq. 5), with magnitude unity inside the domain and zero outside the domain (Fig. 1b) [73]. This enables direct integration of field quantities like stress and strain energy density, making it more robust for nonlinear materials and complex geometries. Additionally, domain integration allows for better handling of plasticity effects, and it improves the numerical stability and accuracy, especially in finite element analysis [74].

EDI is typically coupled with the virtual crack extension (VCE) method that employs Cartesian coordinates aligned with the assumed crack propagation direction. The VCE method then calculates the $\:{J}_{k}$ -integral with the VCE in $\:{x}_{k}$ direction. For single defect problems, physically, the two components of the $\:{J}_{k}$ vectors represent unique energy releases induced by the crack movements along the $\:{x}_{1}$-axis and the $\:{x}_{2}$-axis [16, 75]. Further details on the significance of the VCE direction are discussed in the first case study in section ‎4.1.

Thus, a 3D formulation that uses the EDI and VCE will take the following form:

5 $$\begin{aligned} {J_k} & =\mathop \smallint \limits_{{\mathrm{A}}} \left( {{\sigma _{ij}}{u_{i,k}} - {W}{\delta _{kj}}} \right)\frac{{\partial {q_k}}}{{\partial {x_j}}}dA,\:\: k =1,2 \\ \end{aligned} $$

where $\:dA$ is a surface element on a vanishingly small tubular surface enclosing the crack tip.

To simplify the analysis and efficiently compute the $\:{J}_{k}$-integral without the need to use a shape function to remap element nodes or any other aspects of the finite element method; the VCE direction $\:{x}_{1}\:$is chosen to be parallel to the crack (in practical terms, this can require remapping of experimental data to satisfy this condition so the crack is parallel to the *X*-axis), and the crack tip is located centrally in a square map with an equally spaced grid (typical of experimental measurement) with the measurement points as nodes (Fig. 1b). This simplifies the $\:{J}_{k}$-integral formulation to:

6 $$\begin{aligned} {J_k} & =\mathop \sum \limits_{{j=1}}^{2} \left( {\mathop \sum \limits_{{i=1}}^{3} {\sigma _{ij}}{u_{i,{\mathrm{k}}}} - W{\delta _{kj}}} \right)\frac{{\partial q}}{{\partial {x_j}}}dA, \:\:k =1,2 \\ \end{aligned} $$

### Numerical evaluation of the *M*-Integral

Budiansky and Rice [16] introduced the *M*-integral as the configurational force of multiple or densely distributed defects in inhomogeneous material, as the assessment of the ‘global’^3^ or effective damage state [76–78]. For example, it can be used where there is a defect in a material expanding in multiple directions, contrary to the $\:{J}_{k}$-integral, which evaluates the energy release associated with a singular defect extension. This makes the *M*-integral suitable for multiple and single defects, such as embedded cracks or dislocations pile-up.

As a domain integral, the *M*-integral is formulated as in Eq. 7, which can be simplified when using a regularised mesh to Eq. 8.

7 $$\begin{aligned} {M_k} & =\mathop \smallint \limits_{{\mathrm{A}}} \left( {{\sigma _{ij}}\:{u_{i,k}}\:{x_k} - W\:{\delta _{kj}}\:{x_k}} \right)\frac{{\partial {q_1}}}{{\partial {x_j}}}dA,\:\: k =1,2 \\ \end{aligned} $$

8 $${M_k}=\mathop \sum \limits_{{j=1}}^{2} \left( {\mathop \sum \limits_{{i=1}}^{3} {\sigma _{ij}}{u_{i,{\mathrm{k}}}}{x_k} - W{\delta _{kj}}{x_j}} \right)\frac{{\partial {q_1}}}{{\partial {x_j}}}dA$$

### Numerical evaluation of the stress intensity factors

SIFs are essential in linear elastic fracture mechanics to characterise the magnitude of the crack-tip stress fields, which influence the crack’s growth direction and rate [32], but SIFs can also be used with other stress raisers [28, 79]. Since the scalar energy integral cannot distinguish between different loading modes, mode-decoupling techniques are needed to calculate the SIFs, i.e., tensile/compression mode I, in-plane shear mode II, and out-of-plane shear mode III.

We have developed a mode-decoupling technique that introduces an auxiliary displacement gradient ($\:{u}_{i,j}$) field to decompose the total displacement gradient field into its symmetric (mode I), in-plane skew-symmetric (mode II), and out-of-plane skew-symmetric (mode III) components. It is achieved by overlaying the auxiliary field onto the total field, mirrored along the VCE vector that is aligned with the $\:{x}_{1}$-axis, as described in Eq. (6).

9 $$ \begin{aligned} u_{{i,j}} & = u_{{i,j}}^{I} + u_{{i,j}}^{{II}} + u_{{i,j}}^{{III}} \\ u_{{i,j}}^{I} & = \frac{1}{2}\left( {\begin{array}{*{20}c} {u_{{1,1}} + \bar{u}_{{1,1}} } & {u_{{1,2}} - \bar{u}_{{1,2}} } & {u_{{1,3}} + \bar{u}_{{1,3}} } \\ {u_{{2,1}} - \bar{u}_{{2,1}} } & {u_{{2,2}} + \bar{u}_{{2,2}} } & {u_{{2,3}} - \bar{u}_{{2,3}} } \\ {u_{{3,1}} + \bar{u}_{{3,1}} } & {u_{{3,2}} - \bar{u}_{{3,2}} } & {u_{{3,3}} + \bar{u}_{{3,3}} } \\ \end{array} } \right) \\ u_{{i,j}}^{{II}} & = \frac{1}{2}\left( {\begin{array}{*{20}c} {u_{{1,1}} - \bar{u}_{{1,1}} } & {u_{{1,2}} + \bar{u}_{{1,2}} } & 0 \\ {u_{{2,1}} + \bar{u}_{{2,1}} } & {u_{{2,2}} - \bar{u}_{{2,2}} } & 0 \\ 0 & 0 & {u_{{3,3}} - \bar{u}_{{3,3}} } \\ \end{array} } \right) \\ u_{{i,j}}^{{III}} & = \frac{1}{2}\left( {\begin{array}{*{20}c} 0 & 0 & {u_{{1,3}} - \bar{u}_{{1,3}} } \\ 0 & 0 & {u_{{2,3}} + \bar{u}_{{2,3}} } \\ {u_{{3,1}} - \bar{u}_{{3,1}} } & {u_{{3,2}} + \bar{u}_{{3,2}} } & 0 \\ \end{array} } \right) \\ \end{aligned} $$

For deformation gradient ($\:F$) field, the auxiliary field becomes:

10 $$\begin{aligned} u_{{i,j}} & = \sum\limits_{m} {F_{{ij}}^{m} - \delta \:_{{ij}}^{m} } ,\:\:i,j = {\mathrm{1,2}},3,\:\:m = I,II,III \\ u_{{i,j}}^{I} & = \frac{1}{2}\left( {\begin{array}{*{20}c} {F_{{11}} + \bar{F}_{{11}} - 2\:} & {F_{{12}} - \bar{F}_{{12}}} & {F_{{13}} + \bar{F}_{{13}} } \\ {F_{{21}} - \bar{F}_{{21}} } & {F_{{22}} + \bar{F}_{{22}} - 2} & {F_{{23}} - \bar{F}_{{23}} } \\ {F_{{31}} + \bar{F}_{{31}} } & {F_{{32}} - \bar{F}_{{32}} } & {F_{{33}} + \bar{F}_{{33}} - 2} \\ \end{array} } \right) \\ u_{{i,j}}^{{II}} & = \frac{1}{2}\left( {\begin{array}{*{20}c} {F_{{11}} - \bar{F}_{{11}} } & {F_{{12}} + \bar{F}_{{12}} } & 0 \\ {F_{{21}} + \bar{F}_{{21}} } & {F_{{22}} - \bar{F}_{{22}} } & 0 \\ 0 & 0 & {F_{{33}} - \bar{F}_{{33}} } \\ \end{array} } \right) \\ u_{{i,j}}^{{III}} & = \frac{1}{2}\left( {\begin{array}{*{20}c} 0 & 0 & {F_{{13}} - \bar{F}_{{13}}} \\ 0 & 0 & {F_{{23}} + \bar{F}_{{23}} } \\ {F_{{31}} - \bar{F}_{{31}} } & {F_{{32}} + \bar{F}_{{32}} } & 0 \\ \end{array} } \right) \\ \end{aligned} $$

This derivation deviates from previous formulations [70, 72, 80, 81] based on the stress and strain field decomposition, and not the full-field displacement or deformation gradient. Additionally, including $\:{u}_{\mathrm{1,3}}$, $\:{u}_{\mathrm{2,3}}$, $\:{u}_{\mathrm{3,1}}$, $\:{u}_{\mathrm{3,2}}$ and $\:{u}_{\mathrm{3,3}}$​ in the mode I decomposition and the asymmetrical part of $\:{u}_{\mathrm{3,3}}$ in mode II decomposition accounts for out-of-plane displacement gradients associated with the crack opening and in-plane shear, respectively. While classical mode I is defined by normal separation in the crack plane, real-world specimens exhibit non-negligible out-of-plane effects due to Poisson contraction, surface constraints, and front curvature, especially in ductile materials investigated using stereo-DIC. These components capture the symmetric part of the displacement field in the thickness direction and improve accuracy in quantifying opening-mode deformation under mixed loading conditions.

Note that while this decomposition has been validated against analytical mixed-mode solutions (see Sect. 4.1), its sensitivity to non-ideal out-of-plane effects (e.g., Poisson contraction, curvature-induced distortion, or optical artefacts) depends on the quality and nature of the measurement technique. Quantitative assessment of such artefacts is beyond the scope of this study and has been addressed in work involving uncertainty quantification in HR-EBSD [82–84], DIC [85, 86], or DVC [87–89] measurements.

In order to calculate the strain energy density, $\:\mathrm{W}$; the mode-specific Cauchy’s strain tensor ($\:\varepsilon\:$) is calculated as in Eq. (10).

11 $$\:{\varepsilon}_{ij}^{m}\approx\:\frac{1}{2}\left({u}_{i,j}^{m}+{u}_{j,i}^{m}\right),\:\:m=I,II,III$$

The mode-specific stress ($\:{\sigma}_{ij}^{m}$) is calculated from the strains using the material’s mechanical properties. Then, from each decomposed field, the mode-specific $\:{J}_{1}^{I+II+III}$ or the *J*-integral can be calculated and related to the specific SIFs, as described in Eq. 12 for the example of an isotropic elastic material with Young’s modulus ($\:E$), shear modulus ($\:\mu\:$) and Poisson’s ratio ($\:\nu\:$). For anisotropic elastic materials, the eqvalent $\:E$, $\:\mu\:$ and $\:\nu\:$ can be estimated [90].

12 $$\begin{aligned} K_{I} & = \sqrt {E^{{\prime \:}} J_{1}^{I} } \\ K_{{II}} & = \sqrt {E^{{\prime \:}} J_{1}^{{II}} } \\ K_{{III}} & = \sqrt {2\mu \:J_{1}^{{III}} } \\ E^{\prime } & = \left\{ {\begin{array}{*{20}c} {\frac{E}{{1 - v^{2} }}{\mkern 1mu} \:\:{\text{for plane strain}}} \\ {E{\mkern 1mu} \:\:\:{\text{for plane stress}}} \\ \end{array} } \right., \\ \mu & = \frac{E}{{2\left( {1 + v} \right)}} \\ \end{aligned} $$

13 $$\:{J}_{1}={J}_{1}^{I}+{J}_{1}^{II}+{J}_{1}^{III},\:\:{K}_{\mathrm{e}\mathrm{f}\mathrm{f}}=\sqrt{{J}_{1}{E}^{{\prime\:}}}$$

13)

The effective stress intensity factor $\:{K}_{\mathrm{e}\mathrm{f}\mathrm{f}}$ is calculated similarly by the summation of the mode-specific $\:{J}_{1}^{I+II+III}$ as in Eq. 13, or from the $\:{J}_{1}$ calculated directly from the original field; however, some difference between $\:{J}_{1}^{I+II+III}$ and $\:{J}_{1}$ is expected due to the superimposed auxiliary field for mode decoupling (see Supplementary information: B).

## Structure of the toolbox

**Fig. 2:**
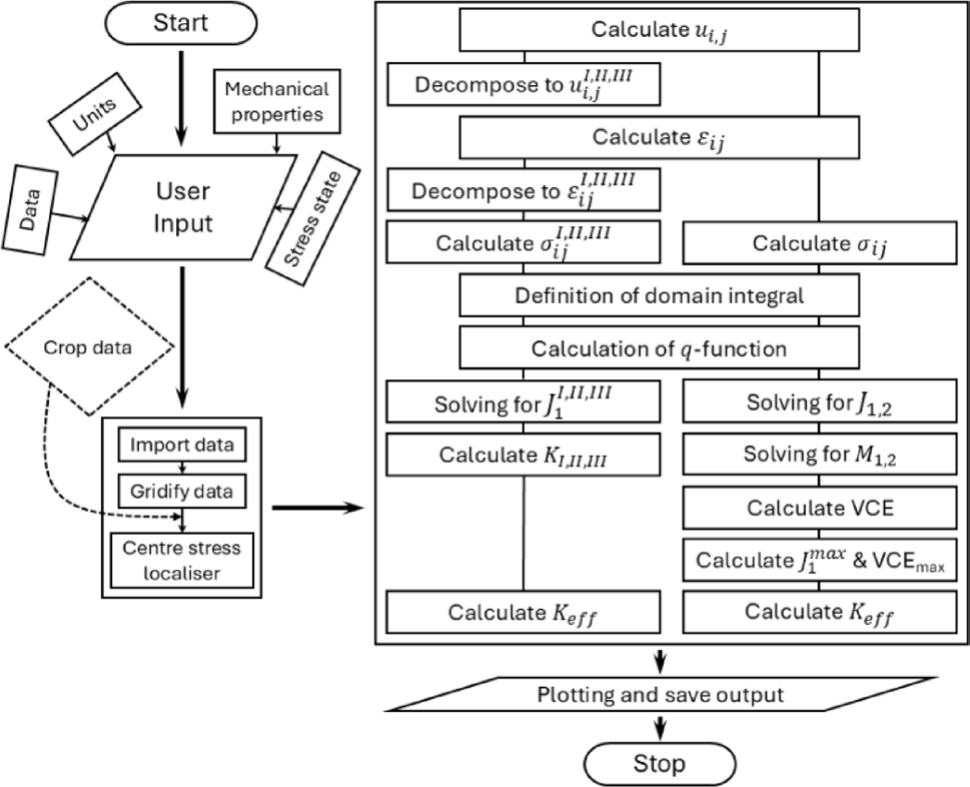
Workflow of the computational toolbox for extracting configurational forces and stress intensity factors. Flowchart illustrating the key modules of the MATLAB-based toolbox, including data input, preprocessing, invariant integral calculations (*J*- and *M*-integrals), mode decomposition, and post-processing. Solid arrows represent the core computational pipeline, while dashed lines indicate optional or user-controlled functionalities. The workflow accommodates both 2D and 3D displacement, or displacement/deformation gradient data and supports materials with isotropic, anisotropic, or elastoplastic behaviour

The toolbox, implemented in MATLAB, calculates the configurational forces, namely the *J*-and *M*-integral, and then the mixed-mode SIFS from a displacement or displacement/deformation gradient field. The displacement field is typically measured using 2D in-plane digital image correlation (DIC), 3D-stereo DIC where two cameras measure both in-plane and out-of-plane [91], or 3D digital volume correlation (DVC) [92–94]. The displacement/deformation gradient field can be obtained using micro-diffraction techniques like high-resolution electron backscatter diffraction (HR-EBSD) [95, 96] or differential-aperture X-ray microscopy (DAXM) [97]. The toolbox is organised into several modules that handle user input, data pre-processing, integral and SIF calculations, and post-processing (Fig. 2).

The toolbox requires the user to input the directory of the data, which can be 2D or 3D-stereo displacement data, 2D sliced 3D data, or displacement/deformation gradient data. The user also needs to input the stress state, (typically, plane stress for surface measurement and plane strain for measurement from confined deformation), the measurement length units (meters, millimetres, micrometres, or nanometres), and the material’s mechanical properties, which can be an isotropic or anisotropic^4^ elastic material, or an elastoplastic material that follows the Ramberg–Osgood relationship^5^ [98].

The input data can be uploaded in any format that MATLAB can read. For 2D displacement measurements, the spatial X and Y coordinates for the displacement $\:{U}_{x}$ in X and displacement $\:{U}_{y}\:$in measurement points is needed. 3D-stereo displacement measurements have the additional out-of-plane displacement, with Z spatial coordinates that should equal zero, as such measurements are typically taken at a surface that is effectively flat. For 2D displacement/deformation gradient measurement, along with the measurement points coordinates, the 6 displacement/deformation gradient in-plane components are required. This becomes 9 components for 3D-stereo displacement/deformation gradient measurement.

Poor quality or uncertain data may arise for various reasons, including material discontinuities [99, 100] like grain boundaries or crack faces. These must be removed from the data and replaced with NaNs (not a number). The input deformation field must also be restricted to one defect or agglomeration of defects that do not strongly interact with other defects (including grain boundaries), as that will affect the path independence of the invariant integrals [101–103].

The toolbox requires the data to be a regularised mesh or measurement points with a constant step size, which is commonplace for most experimental methods. A comparably coarse step size is sufficient for invariant-integral calculations, with no refinement near the defect being needed [104]. However, if this is not the case, once the data is uploaded, it will be automatically mapped into equally spaced grids, i.e., a square grid with four nodes/data points.

Following the data reading, the user locates the defect. For a crack, that will be the crack tip, and for a dislocation, that will be its core. The user then defines a square around the defect that will be used to calculate the invariant integrals through the equivalent domain integral (EDI) method. The displacement gradient is then calculated and decoupled into $\:{u}_{i,j}^{I}$, $\:{u}_{i,j}^{II}$, $\:{u}_{i,j}^{III}$ for mode I–III. The strain is calculated from the decomposed displacement or deformation gradient, as explained in section ‎2.3. Then, the stress is calculated depending on the material’s stress-strain relationship.

As explained above, the *J*- and *M*-integrals are calculated using the equivalent domain integral (EDI) method coupled with the virtual crack extension (VCE) [73, 105] parallel to the horizon or X-axis, i.e., $\:\theta\:$ = 0° (illustrated in Fig. 1). To explore changes in the VCE direction (or $\:\theta\:$), the field can be transformed using a rotation matrix ($\:{R}_{\theta\:}$) as follows:

14 $$\begin{aligned} u_{{i,j}}^{\theta } & = R_{{z\left( {\theta}\right)}} \:u_{{i,j}} \:R_{{z\left( {\theta} \right)}}^{T} \\ R_{{z\left( {\theta \:} \right)}} & = \left[ {\begin{array}{*{20}c} {{\mathrm{cos}}\left( \theta \right)} & { - {\mathrm{sin}}\left( \theta \right)} & 0 \\ {{\mathrm{sin}}\left( \theta \right)} & {{\mathrm{cos}}\left( \theta \right)} & 0 \\ 0 & 0 & 1 \\ \end{array} } \right] \\ \end{aligned} $$

While the toolbox requires specifying a defect location and VCE direction, these parameters can be derived directly from the displacement field. For example, crack tips can be identified through field gradient analysis or phase congruency filters [106–109], and the VCE direction can be iteratively refined by comparing $\:{J}_{1}$​ and $\:{J}_{2}$​ values across directions. This removes the need for predefined geometry or load assumptions and allows the analysis to adapt to arbitrary crack shapes and propagation paths.

**Fig. 3:**
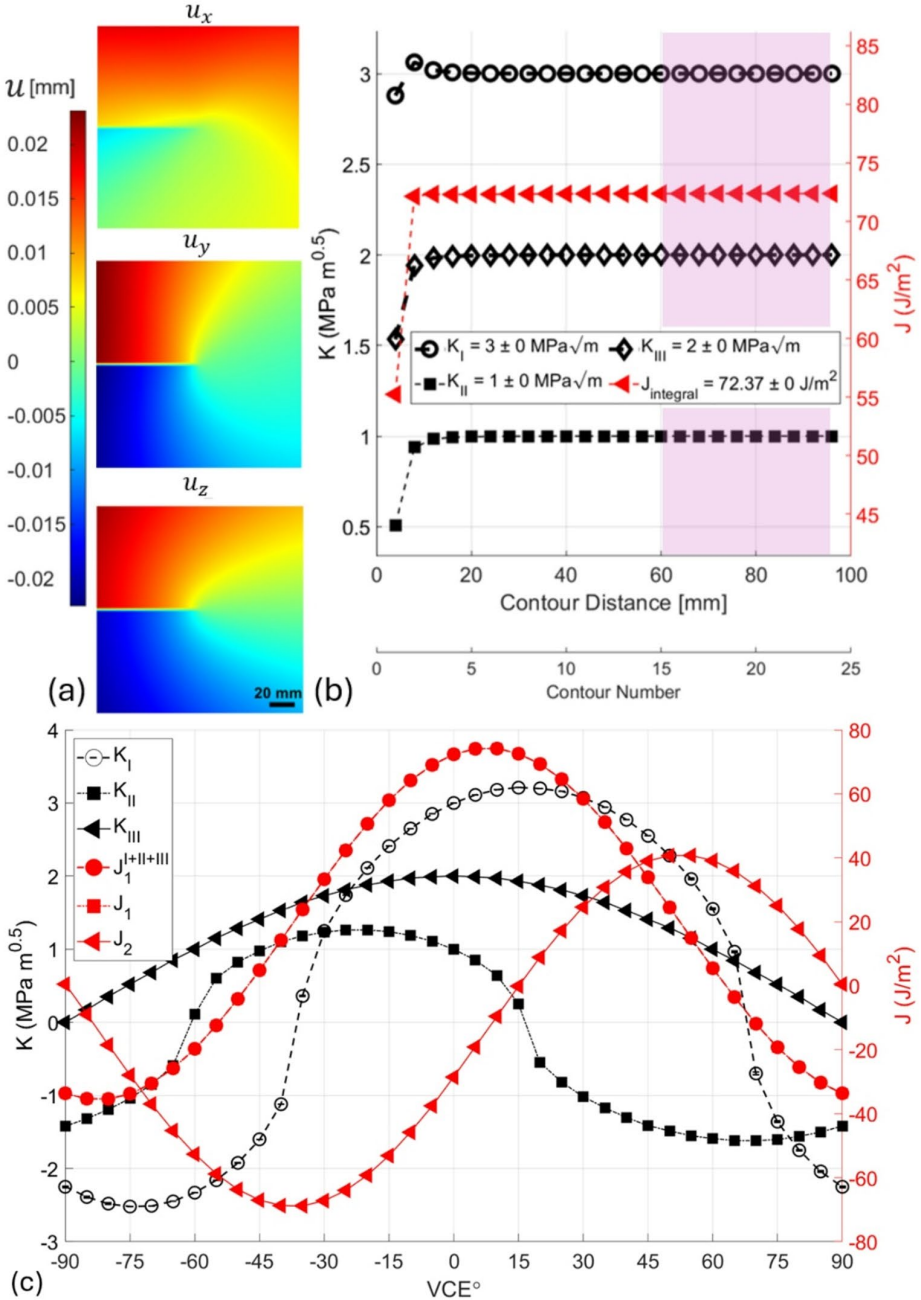
Validation of the toolbox using synthetic displacement fields for a stationary mixed-mode crack: **a** Synthetic displacement field generated using Westergaard’s solution for a stationary crack under mixed-mode loading: (mode I: 3 MPa m^0.5^, mode II: 1 MPa m^0.5^, and mode III: 2 MPa m^0.5^), assuming plane stress conditions. The field of view spans 200 × 200 mm² with a crack tip at the centre. **b** Calculated *J*-integral and decomposed SIFs for modes I–III as a function of domain expansion using the EDI method. Convergence trends are shown as the domain extends away from the crack tip, demonstrating accurate recovery of input values. **c** The sensitivity of the $\:{J}_{k}$-integral and SIF components to the direction of the VCE, varied from − 90° to + 90° in 5° increments. $\:{J}_{1}$ directly calculated from the field, and $\:{J}_{1}^{I+I+III}$ calculated from summing the mode-specific $\:{J}_{1}$ overlap

Next, the user selects the number of expanded domains to consider by inspecting the *J*-integral’s convergence, since convergence may fail when the integration domain extends into peripheral stress fields. As discussed in prior research [101–103], contributions from surrounding gradient fields can disrupt path independence unless fully enclosed within the integration domain. The mean and standard deviation of the invariant integrals and SIFs are calculated where stable convergence has been achieved (shaded pink in Fig. 3b).

## Illustrative examples

To demonstrate the versatility of the toolbox, below we present several case studies where we used a (1) 3D-stereo synthetic displacement field for a mixed mode crack to validate the toolbox, (2) high-resolution electron backscatter diffraction (HR-EBSD) displacement gradient field around a micro-crack in a single crystal ceramic, (3) high-resolution transmission Kikuchi diffraction (HR-TKD) displacement gradient field mapped around a dislocation in an anisotropic metal, and (4) 3D displacement field obtained by digital volume correlation (DVC) of computed X-ray tomographs of a fatigue crack with complicated geometry.

### Case study 1: analytical field benchmarking

A mixed-mode crack displacement field that has a mode I stress intensity factor ($\:{K}_{I}$) of 3 MPa m^0.5^, mode II ($\:{K}_{II}$) of 1 MPa m^0.5^, and mode III ($\:{K}_{III}$) of 2 MPa m^0.5^ was created using the Westergaard analytical solution [110], with the assumption of plane stress conditions (Eq. 12 to 15). The material mechanical properties were defined as linear isotropic with Young’s modulus ($\:E$) of 210 GPa and Poisson’s ratio ($\:v$) of 0.3. The field of view was 200 × 200 mm^2^, comprised of 4 × 4 mm^2^ square elements with the crack tip at the centre.

15 $$ \begin{aligned} u_{x} & = \frac{{K_{I} }}{{2\mu }}\sqrt {\frac{r}{{2\pi }}} {\mathrm{cos}}\left( {\frac{\theta }{2}} \right)\left[ {k - 1 + 2{\mathrm{sin}}^{2} \left( {\frac{\theta }{2}} \right)} \right] \\ & + \frac{{K_{{II}} }}{{2\mu }}\sqrt {\frac{r}{{2\pi }}} {\mathrm{sin}}\left( {\frac{\theta }{2}} \right)\left[ {k + 1 + 2{\mathrm{cos}}^{2} \left( {\frac{\theta }{2}} \right)} \right] \\ \end{aligned} $$

16 $$ \begin{aligned} u_{y} & = \frac{{K_{I} }}{{2\mu }}\sqrt {\frac{r}{{2\pi }}} {\mathrm{cos}}\left( {\frac{\theta }{2}} \right)\left[ {k + 1 - 2{\mathrm{cos}}^{2} \left( {\frac{\theta }{2}} \right)} \right] \\ & - \frac{{K_{{II}} }}{{2\mu }}\sqrt {\frac{r}{{2\pi }}} {\mathrm{cos}}\left( {\frac{\theta }{2}} \right)\left[ {k - 1 - 2{\mathrm{sin}}^{2} \left( {\frac{\theta }{2}} \right)} \right] \\ \end{aligned} $$

17 $$\:{u}_{z}=\frac{2{K}_{III}}{\mu\:}\sqrt{\frac{r}{2\pi\:}}\mathrm{sin}\left(\frac{\theta\:}{2}\right)$$

18 $$\:k=\frac{3-v}{1+v}$$

The mixed-mode SIFs computed by the toolbox from this synthetic displacement field matched with the prescribed inputs mode I–III (Fig. 3b). The presence of highly localised fields near the crack tip detrimentally influences the initial convergence of the integral; however, convergence stabilises as the integration domain expands, which is expected due to the crack-tip singularity [110]. At very small radii, numerical noise and steep gradients affect stability. As the domain expands to enclose the energy release zone, the configurational force estimate stabilises. While no universal criterion applies, in practice, the measured displacement or gradient field must span a sufficiently broad region around the crack or defect to allow such convergence, and the region immediately adjacent to the singularity should be excluded from the integration contour.

Additional factors affecting convergence, such as noise and uncertainty in the crack position, are examined further in the Supplementary information: A. In addition to the analytical benchmark, we validated the decomposition against a finite element displacement field generated in Abaqus. The recovered SIFs closely matched the known applied values, further supporting the generalisability of the approach beyond idealised cases (see Supplementary Information: B).

The VCE direction was then varied from − 90° to 90° with a step size of 5°, by transforming the input field, to study the effect of the assumed VCE direction on the calculations. As shown in Fig. 3c, the energy release and mechanical conditions at the crack tip change with direction because if the crack propagates in the assumed VCE direction, the magnitudes of the modes of the mechanical conditions differ at the tip. This variation associated with the VCE might be used in fracture experiments to test criteria that describe mixed-mode crack propagation, which is still a point of contention [111–114].

Energy-based criteria that use the energy-based path integrals or strain energy density appear to hold great potential for both elastic and elastoplastic materials [112–115], especially for dynamic loading [116]. For the current synthetic data, and using the Newton-Raphson method for a solution that maximises $\:{J}_{1}$, we found that $\:{\theta}_{max}$ is at 7.44°, which results in a $\:{J}_{1}$ of 74.33 J m^− 2^ and $\:{J}_{2}$ of -14.47 J m^− 2^, with the SIFs for this direction being 3.15 MPa m^0.5^ for mode I, 0.76 MPa m^0.5^ for mode II, and 1.98 MPa m^0.5^ for mode III. Thus, under these conditions, the crack is expected to slightly deflect (or kink) to maximise its energy [117, 118].

Given the strong sinusoidal relationship between the $\:{J}_{1}$, $\:{J}_{2}$ and the VCE, the outputs can be fitted by Eq. 16, without considering the offset via a rotation matrix, but we encourage the reader to consider formulating a more elegant solution that considers the offset.

19 $$\begin{aligned} J_{k}^{\theta } & = R_{{k,\theta }} \,J_{k}, \:\: k = {\mathrm{1,2}} \\ R_{\theta } & = \left[ {\begin{array}{*{20}c} {cos\theta } & { - sin\theta } \\ {sin\theta } & {cos\theta } \\ \end{array} } \right] \\ \end{aligned}$$

### Case study 2: Mixed-mode micro-crack in a brittle ceramic (6 H-SiC)

**Fig. 4:**
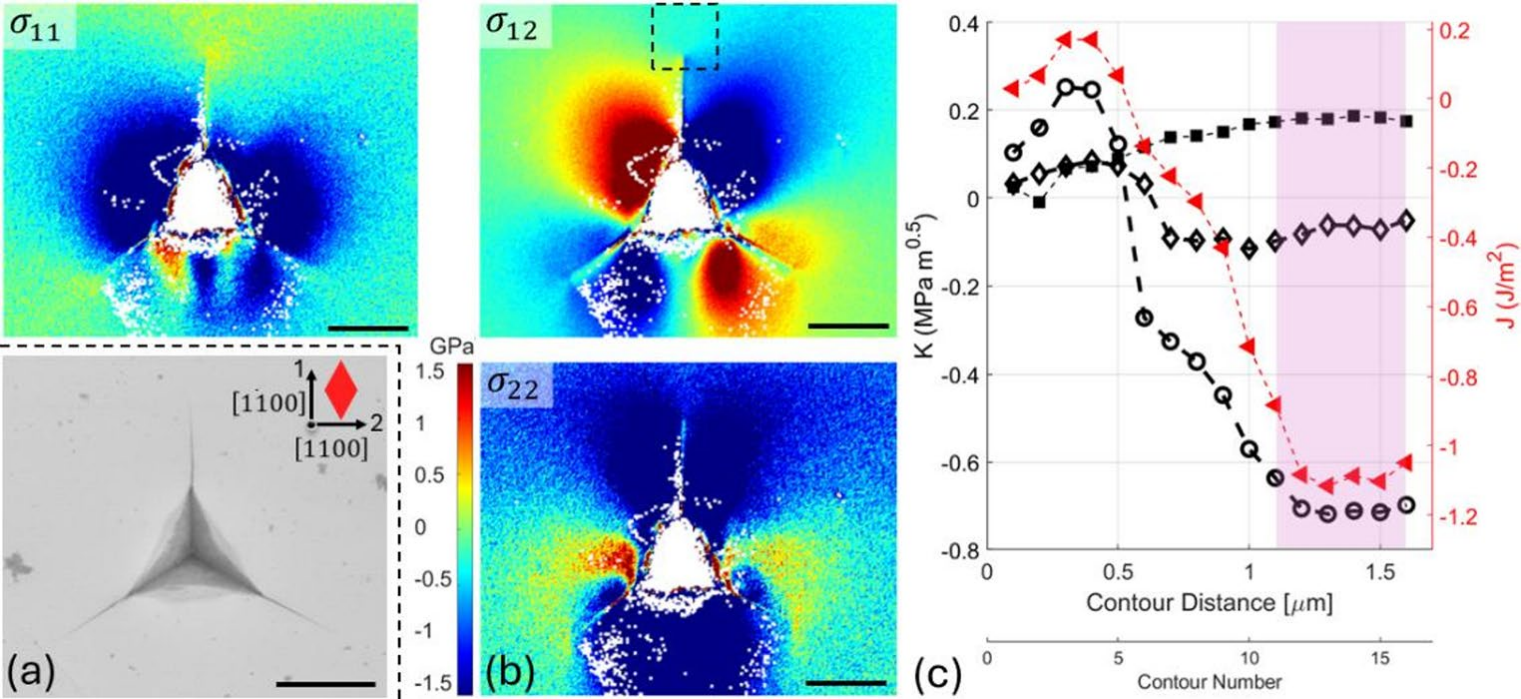
**a** SEM image of the nano-indented (0001) 6 H-SiC single crystal with the axes and the crystal orientation. **b** The in-plane stress components calculated from HR-EBSD using anisotropic Hooke’s law and the (0001) 6H-SiC material anisotropic stiffness matrix, with ε_33_ strain calculated with plane stress assumption. **c**)*J*-integral and SIFs convergence for the crack dashed in b. The scale bar is 5 μm

Measuring microscale fracture toughness is vital for assessing the durability of brittle coatings and hard surfaces under stress. Microscale fracture toughness, particularly for brittle coatings and hard surfaces, is often estimated using indentation-induced cracking and empirical equations based on assumptions about crack geometry and loading conditions. Here, we used data from Leide et al. [119] where a stress-free (0001) 6 H-SiC single crystal was nano-indented with a Berkovich tip (Fig. 4a), employing the continuous stiffness method (CSM) with 2 nm harmonic displacement at 45 Hz, and a 0.05 s^− 1^ strain rate. EBSD was performed on a Zeiss Merlin FEG-SEM using a Bruker detector, capturing high-resolution diffraction patterns at 100 nm steps and 50 ms per pixel at the 1-µm-deep indent. Via the CrossCourt software, patterns were processed using FFT-based cross-correlation (i.e., HR-EBSD) across 40 overlapping regions of interest to compute the elastic displacement gradient tensors.

The elastic displacement gradient field at one of the cracks (dashed box in Fig. 4b) caused by the indent was analysed with the toolbox to assess the conditions at the crack tip. The crack is parallel to $\:\left[1\stackrel{-}{1}00\right]$, which is the trace of the expected $\:\left(11\stackrel{-}{2}0\right)$ cleavage plane. The VCE direction was assumed parallel to the crack, with the integration domain expanding at 100 nm per domain from the crack tip. Stable convergence, pink shaded area in Fig. 4c, was achieved 1.2 μm ahead of the crack tip, revealing a mixed mode crack field with mode I of -0.70 ± 0.03 MPa m^0.5^, mode II of 0.18 ± 0.17 MPa m^0.5^, mode III of -0.07 ± 0.02, and *J*-integral of -1.05 ± 0.08 J m^− 2^. The signs of mode II (in-plane shear) and mode III (out-of-plane shear) components depend on the nodal configuration and symmetry in the field and do not reflect physically meaningful directionality. In contrast, the sign of mode I (tensile vs. compressive) is physically significant.

Cracking during sharp indentation of hard materials arises from the highly localised stress fields generated beneath the indenter. The geometry of the Berkovich tip produces significant triaxial tensile stresses at and around the contact zone during indentation [120], which can exceed the material’s fracture toughness [121, 122]. In 6H-SiC, which exhibits limited plasticity and is brittle, these stresses initiate radial cracks along the weak cleavage planes, i.e., the $\:\left\{11\stackrel{-}{2}0\right\}$ prismatic planes in the orientation of this experiment. Upon unloading, the interaction between the indentation-induced plastic zone and the surrounding elastic matrix leads to a residual stress field. Spallation may occur when the residual stresses are sufficiently high to cause lateral fractures on shallow subsurface cleavage planes (e.g. the basal plane in the orientation of this experiment).

Here, no spallation was observed, and the stress map (Fig. 4b), which was measured after unloading, shows the elastic recovery generated compressive residual stresses. The negative *J*-integral and mode I SIF both show that the radial crack is effectively clamped shut after unloading. The mode II and III SIFs show that the residual stress also causes a small, but measurable, shear loading of the crack. Although the indentation-induced crack is not in critical condition, the toolbox can be used to evaluate the fracture toughness by measuring the mixed-mode crack tip field of a static crack at the critical condition for crack propagation, as done in [123] for cleavage in single-crystal silicon.

### Case study 3: edge dislocation in anisotropic tungsten

**Fig. 5:**
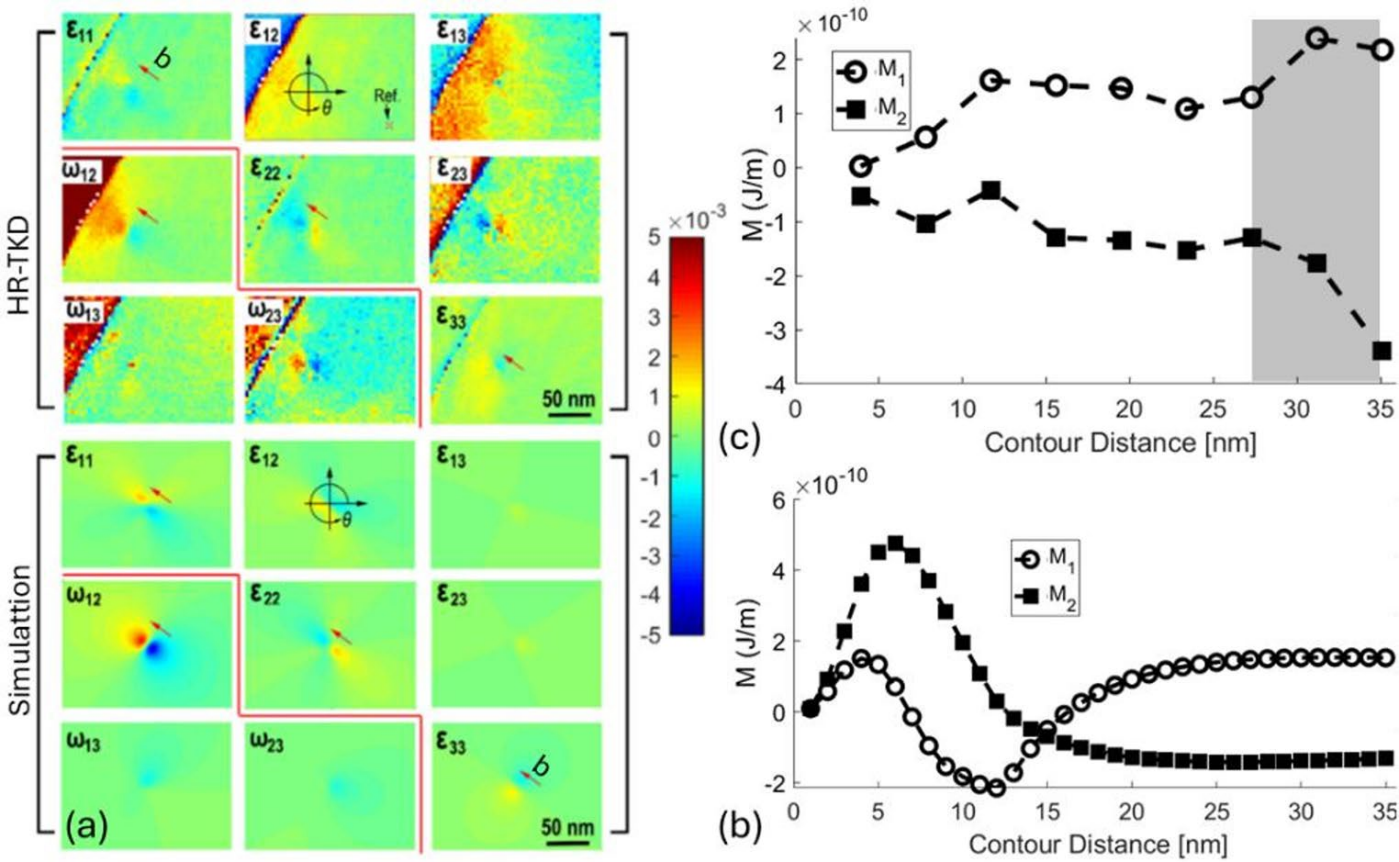
Application of the toolbox to experimental and simulated dislocation fields in anisotropic tungsten. **a** High-resolution transmission Kikuchi diffraction (HR-TKD) and simulated maps of the three-dimensional lattice strain tensor and lattice rotations around an edge dislocation in pure tungsten (reproduced from [128]). **b** *M*-integral convergence for the **b** simulated and **c** experimental dislocation fields, with shaded area, denotes the loss of convergence in the HR-TKD field beyond 15.6 nm due to interference from the grain boundary (top left in a) and measurement distortions near boundaries

Dislocations are one of the most important defects in solids, since they influence the properties of crystals, not only the mechanical, but also the electric, magnetic, optic, and semi-conducting properties [124–126] as well as the growth of crystals [127]. Here, we use the data collected by Yu et al. [128], where they employed high-resolution transmission Kikuchi diffraction (HR-TKD) to map the full elastic displacement gradient induced by a single edge dislocation in pure tungsten (Fig. 5a). Yu et al. then compared the measured field to forward calculations based on an elastically isotropic dislocation model and its Burgers vector [129], demonstrating a high degree of agreement and validating the capability of HR-TKD for resolving lattice distortions at the nanoscale.

The input to the toolbox was the full 3D displacement gradient tensor obtained from HR-TKD, including all nine components, mapped onto a regularised measurement grid. We then transformed the measured displacement gradient field so that the VCE had the same direction as the $\:\left[1\stackrel{-}{1}1\right]a/2$ Burgers vector. The anisotropic stiffness matrix for pure body-centred cubic tungsten ($\:{C}_{11}=522.39,\:{C}_{44}=160.83,\:{C}_{12}=204.37$ in GPa) was transformed to the frame of reference using a rotation matrix ($\:{R}_{{\phi}_{1},{\Phi},{\phi}_{2}}$) constructed from the Euler angles ($\:{\phi}_{1},{\Phi},{\phi}_{2}$) of the reference pattern (measured remote from the defect) as in Eq. (17) [130].

20 $$R_{{\phi_{1} ,\Phi,\phi_{2} }} = \left[ {\begin{array}{*{20}c} {{\mathrm{cos}}\phi_{1} \:{\mathrm{cos}}\phi_{2} - {\mathrm{cos}}\Phi \:{\mathrm{sin}}\phi_{1} \:{\mathrm{sin}}\phi_{2} } & {{\mathrm{cos}}\Phi \:{\mathrm{cos}}\phi_{1} \:{\mathrm{sin}}\phi_{2} + {\mathrm{sin}}\phi_{1} \:{\mathrm{cos}}\phi_{2} } & {{\mathrm{sin}}\Phi \:{\mathrm{sin}}\phi_{2} } \\ { - {\mathrm{cos}}\phi_{1} \:{\mathrm{sin}}\phi_{2} - {\mathrm{cos}}\Phi \:{\mathrm{sin}}\phi_{1} \:{\mathrm{cos}}\phi_{2} } & {{\mathrm{cos}}\Phi \:{\mathrm{cos}}\phi_{1} \:{\mathrm{cos}}\phi_{2} - {\mathrm{sin}}\phi_{1} \:{\mathrm{sin}}\phi_{2} } & {{\mathrm{sin}}\Phi \:{\mathrm{cos}}\phi_{2} } \\ {{\mathrm{sin}}\Phi \:{\mathrm{sin}}\phi_{1} } & { - {\mathrm{sin}}\Phi \:{\mathrm{cos}}\phi_{1} } & {{\mathrm{cos}}\Phi} \\ \end{array} } \right] $$

By using the rotation matrix, the anisotropic elastic stiffness matrix, $\:{C}_{ijkl},\:$can be mapped from the reference crystal coordinate system to the local coordinate system as below [127, 131]:

21 $$\:{C}_{ijkl}^{*}\:={T}_{\sigma\:}^{-1}\:{C}_{ijkl}\:{T}_{\varepsilon\:}\:$$

where the stress and strain transformation tensor, $\:{T}_{\sigma\:}$ and $\:{T}_{\epsilon\:}$, account for how stresses and strain change under coordinate rotation, respectively, and they are calculated from the rotation matrix as follows:

22 $$ T_{\sigma } = \left[ {\begin{array}{*{20}c} {R_{{11}}^{2} } & {R_{{12}}^{2} } & {R_{{13}}^{2} } & {2R_{{12}} R_{{13}} } & {2R_{{11}} R_{{13}} } & {2R_{{11}} R_{{12}} } \\ {R_{{21}}^{2} } & {R_{{22}}^{2} } & {R_{{23}}^{2} } & {2R_{{22}} R_{{23}} } & {2R_{{21}} R_{{23}} } & {2R_{{21}} R_{{22}} } \\ {R_{{31}}^{2} } & {R_{{32}}^{2} } & {R_{{33}}^{2} } & {2R_{{32}} R_{{33}} } & {2R_{{31}} R_{{33}} } & {2R_{{31}} R_{{32}} } \\ {R_{{21}} R_{{31}} } & {R_{{22}} R_{{32}} } & {R_{{23}} R_{{33}} } & {R_{{22}} R_{{33}} + R_{{23}} R_{{32}} } & {R_{{21}} R_{{33}} + R_{{23}} R_{{31}} } & {R_{{21}} R_{{32}} + R_{{22}} R_{{31}} } \\ {R_{{11}} R_{{31}} } & {R_{{12}} R_{{32}} } & {R_{{13}} R_{{33}} } & {R_{{12}} R_{{33}} + R_{{13}} R_{{32}} } & {R_{{11}} R_{{33}} + R_{{13}} R_{{31}} } & {R_{{11}} R_{{32}} + R_{{12}} R_{{31}} } \\ {R_{{11}} R_{{21}} } & {R_{{12}} R_{{22}} } & {R_{{13}} R_{{23}} } & {R_{{12}} R_{{23}} + R_{{13}} R_{{22}} } & {R_{{11}} R_{{23}} + R_{{13}} R_{{21}} } & {R_{{11}} R_{{22}} + R_{{12}} R_{{21}} } \\ \end{array} } \right] $$

23 $$\:{T}_{\varepsilon\:}=\left[\begin{array}{cccccc}{R}_{11}^{2}&\:{R}_{12}^{2}&\:{R}_{13}^{2}&\:{R}_{12}{R}_{13}&\:{R}_{11}{R}_{13}&\:{R}_{11}{R}_{12}\\\:{R}_{21}^{2}\:&\:{R}_{22}^{2}&\:{R}_{23}^{2}&\:{R}_{22}{R}_{23}&\:{R}_{21}{R}_{23}&\:{R}_{21}{R}_{22}\\\:\:{R}_{31}^{2}&\:\:{R}_{32}^{2}&\:{R}_{33}^{2}&\:{R}_{32}{R}_{33}&\:{R}_{31}{R}_{33}&\:{R}_{31}{R}_{32}\\\:2{R}_{21}{R}_{31}&\:2{R}_{22}{R}_{32}&\:{2R}_{23}{R}_{33}&\:{R}_{22}{R}_{33}+{R}_{23}{R}_{32}&\:{R}_{21}{R}_{33}+{R}_{23}{R}_{31}&\:{R}_{21}{R}_{32}+{R}_{22}{R}_{31}\\\:2{R}_{11}{R}_{31}&\:2{R}_{12}{R}_{32}&\:2{R}_{13}{R}_{33}&\:{R}_{12}{R}_{33}+{R}_{13}{R}_{32}&\:{R}_{11}{R}_{33}+{R}_{13}{R}_{31}&\:{R}_{11}{R}_{32}+{R}_{12}{R}_{31}\\\:2{R}_{11}{R}_{21}&\:2{R}_{12}{R}_{22}&\:2{R}_{13}{R}_{23}&\:{R}_{13}{R}_{23}+{R}_{13}{R}_{22}&\:{R}_{11}{R}_{23}+{R}_{13}{R}_{21}&\:{R}_{11}{R}_{22}+{R}_{12}{R}_{21}\end{array}\right]$$

The toolbox used the experimental and simulated dislocation displacement gradient fields to calculate the dislocation’s configurational force, i.e., the *M*-integral. For the high-resolution simulated data, the domain integration started from the dislocation core and expanded gradually with an increment of 1 nm (Fig. 5b). Convergence was achieved once the domain expanded to engulf the dislocation at around 20 nm, with results of $\:{M}_{1}$ = 0.142 ± 0.014 nJ m^−1^ and $\:{M}_{2}$ = -0.138 ± 0.003 nJ m^−1^.

As for the relatively low-resolution experimental data, the domain integration expanded in increments of 3.9 nm, and convergence was achieved at 15.6 nm from the core (Fig. 5c). However, once the domain reached the grain boundary (shaded area in Fig. 5c), it lost convergence as the grain boundary also acts as a stress localiser. More importantly, the displacement gradient field calculated from the Kikuchi patterns near the grain boundaries is unreliable due to the beam volume interaction with both grains near the grain boundary, which causes pattern distortion [132]. The analysis of the experimental observation of the dislocation found $\:{M}_{1}$ = 0.134 ± 0.020 nJ m^−1^ and $\:{M}_{2}$ = -0.137 ± 0.011 nJ m^−1^, which agrees with the simulation.

### Case study 4: 3D displacement field of a fatigue crack

**Fig. 6:**
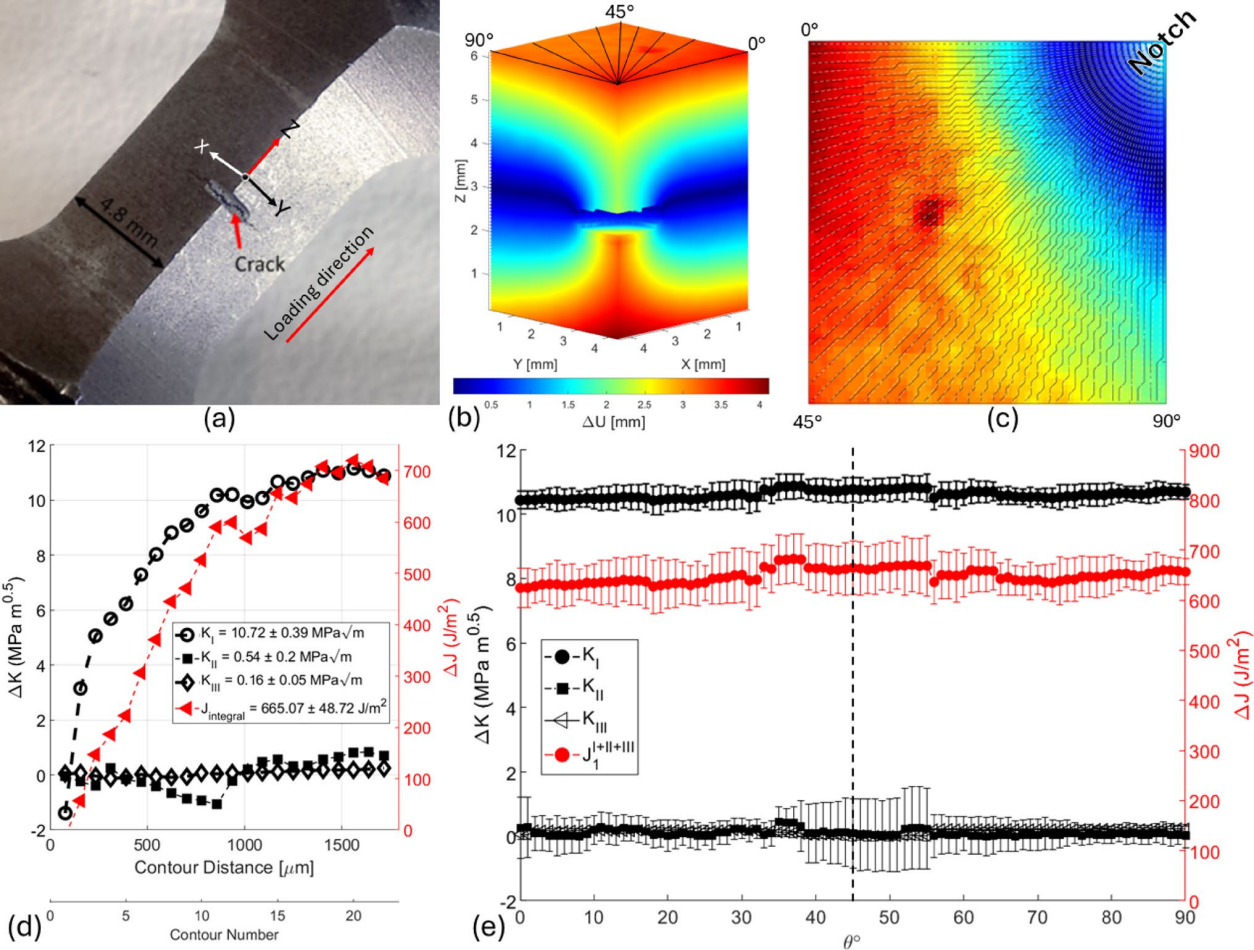
Analysis of a 3D displacement field from a fatigue crack using the computational toolbox. **a** Optical image of a compact tension specimen showing the notch and a fully developed fatigue crack. **b** The three-dimensional displacement field (U_z_), aligned with the loading direction, was measured via DVC after 230,000 loading cycles (P_min_ = 450 N, P_max_ = 4500 N). Displacement field extracted with a 96 × 96 × 96 voxel subset and 75% overlap. **c** Illustration of the radial slicing of the crack front used for virtual crack extension (VCE) analysis, with layers sampled from 0° to 90° along the crack edge at 1° intervals. **d** $\:{J}_{1}^{I,III,III}$ integral and SIFs convergence for the crack front at 33°, showing stable convergence, using the equivalent domain integral method, 1 mm away from the crack tip. **e** Mode I–III and the *J*-integral across the crack front, revealing dominant mode I

Fatigue crack growth under cyclic loading is a critical failure mechanism in structural materials, and understanding the local mechanical conditions around the crack front is essential for predicting durability and lifetime [92–94]. Here, we used an in-situ synchrotron X-ray computed tomography (XCT) dataset from observations of a fatigue crack in nodular cast iron. The data considered here were collected for a well-developed crack at two different loads, after 230,000 loading cycles of P_min_ = 450 N and P_max_ = 4500 N [133]. The three-dimensional displacement field was determined from the image volumes by local DVC using a subset of 96 × 96 × 96 voxels with 75% overlap, yielding high-resolution measurements of the U_x_, U_y_, and U_z_ components. Figure 6a shows the specimen and the notch–crack geometry.

Before using the toolbox, phase congruency filtering of the displacement field was used to isolate the crack region and remove noisy data along the crack faces (Fig. 6b), as DVC struggles near crack faces due to edge effects [99, 100]. Additionally, a reference point was identified and set as the origin to correct for rigid body motions and ensure accurate field interpretation [134]. This ensured that all measured displacements accurately reflected the material’s deformation, free from artefacts caused by sample movement during testing. To examine the radial nature of the field around the curved crack front, the displacement field was sectioned without extrapolation into angular layers from 0° to 90° (Fig. 6c). Each section was analysed as a 2D problem, using the EDI method, aligned with the local crack front direction using linear elastic material properties of $\:E$ = 158 GPa and $\:\nu\:$ = 0.3 [135] and assuming plane strain conditions, as the XCT measurement started 0.3 mm deep in the sample.

Figure 6d shows an example of the convergence of the *J*-integral in the 33° section, where stable integration was achieved after a contour size of 1 mm. The resulting SIFs at 33° indicate a dominant mode I of 10.72 ± 0.39 MPa m^0.5^, with insignificant mode II of 0.54 ± 0.20 MPa m^0.5^ and mode III of 0.16 ± 0.05 MPa m^0.5^. Overall, the variation around the curved crack front (Fig. 6e) shows that the mechanical loading was predominantly mode I at 10.61 ± 0.12 MPa m^0.5^. The average mode I SIF is consistent with the original publication and the observed crack growth rate [133, 136]. The distribution of mode I SIFs along the crack front differs slightly from that reported in the original publication [133], in which the calculated mode I values were distributed more asymmetrically. That analysis, in which it was assumed that the mode III could be neglected, was performed using a finite element-based method in Abaqus. In contrast, the present results were obtained directly from the experimental DVC field using the path-independent integrals implemented in the toolbox to consider all modes. Differences in the assumed crack propagation direction could also affect the analysis, influencing the mode decomposition.

It is important to note that the method worked on this experimental data because the load is predominantly in the opening direction. However, where there is a significant out-of-plane displacement component, direct application of the decomposition method does not allow for stress equilibrium in the far-field of the crack for the case of mode III, which affects the convergence of the $\:{J}_{1}^{III}$-integral and leads to overestimation of mode III [137], as shown in the Supplementary information: C. This is not a limitation of the toolbox itself but reflects the difficulty of resolving equilibrium-consistent mode III fields from DVC data. Users should therefore interpret Mode III values cautiously unless validated through simulation or corrected using inverse modelling techniques. Future work may address this by integrating equilibrium-enforcing corrections [138].

## Discussion

The presented computational toolbox offers a generalisable and robust method for extracting fracture parameters (i.e. configurational forces and mixed-mode stress intensity factors) directly from experimental displacement and gradient fields, without requiring predefined geometry, boundary conditions, or numerical meshing. Its validity and versatility are demonstrated across synthetic, 2D experimental, and 3D datasets, including microcracks in a ceramic, dislocations in anisotropic materials and fatigue cracks in cast iron. The method reliably handles complex defect morphologies and noisy^6^ displacement fields, and it is well-suited for experimental platforms such as DIC, DVC, HR-EBSD and HR-TKD. The accuracy of the extracted stress intensity factors and configurational forces depends directly on the quality of the input field data. The toolbox does not compensate for measurement artefacts, equilibrium violations, or poor resolution; rather, it is designed to extract physical quantities from well-resolved, experimentally consistent displacement or deformation gradient fields. Users should ensure appropriate filtering, denoising, or validation when working with experimental data. Simply put, the method performs well when fed good data; poor-quality input will yield unreliable outputs.

Microscale fracture toughness, particularly for brittle coatings and hard surfaces, is often estimated using indentation-induced cracking and empirical equations based on assumptions about crack geometry [121, 122, 139] and loading conditions [140]. A widely used approach, developed by Lawn et al. [40, 141–143], relates mode I fracture toughness to measurable parameters from Vickers indentation, though variations like Berkovich indentation exist [144]. However, the method suffers from uncertainties [145], such as ambiguous subsurface crack shapes [143], variability in fitting factors [146], and material-specific deformation behaviours, like phase transformations in silicon [147, 148]. These complexities, along with user bias and material anisotropy [149–151], raise concerns about the accuracy and reliability of indentation-based toughness values, leading some to argue that such data should be used only comparatively [152].

The presented computational toolbox is a potential game changer, enabling more rigorous, geometry-independent, and physically meaningful evaluation of fracture toughness at the microscale, overcoming the limitations of empirical indentation models by directly quantifying stress intensity factors and energy release rates from experimentally measured fields. Although the toolbox is general in formulation, the present implementation is grounded in small-strain theory, limiting its immediate applicability to materials such as metals and ceramics where these assumptions hold. Extending the framework to support finite-deformation mechanics would enable its application to soft hyperelastic materials. Recent work [153] offers a promising foundation for incorporating configurational force formulations under large strain, and integrating such capabilities is a key direction for future development.

Nonetheless, for path-independent energy integrals to yield meaningful results, the integration domain must fully enclose the region influenced by the defect. The integral may become path-dependent if the domain fails to capture the localised strain fields. This can occur, for example, near a plastically strained crack tip, as in the presence of significant plastic deformation, the energy integrals may exhibit path dependence until the integration domain fully encompasses the plastic zone [102]. Also, pronounced deformation of microstructurally heterogeneous materials might be an issue, particularly in small-scale experiments or when the defect field interacts with boundaries or neighbouring defects. These factors need to be considered when designing experiments, i.e., microstructure length-scale, field of view and spatial resolution of measurements. Once these are considered, the computational framework presented here provides a robust and generalisable solution, particularly for analysing complex and non-standard defect geometries. Its ability to handle anisotropic and elastoplastic materials^7^, as well as 3D displacement fields, enables the investigation of real-world microstructural damage scenarios.

In addition, incorporating DIC, DVC, and diffraction-based techniques (e.g., HR-TKD, HR-EBSD) bridges the gap between experimental measurements and computational mechanics. By directly processing experimentally acquired local field data, the toolbox eliminates the need for global load measurements or assumptions about boundary conditions. This significantly reduces uncertainties caused by misalignment, residual stresses, or compliance effects commonly affecting conventional approaches [154, 155].

While the toolbox is primarily designed to operate on full deformation or displacement gradient fields, it can also be applied to strain measurements, such as those obtained using X-ray [156] or neutron diffraction [157]. However, as these methods provide only the symmetric strain tensor, they do not capture the full deformation gradient, which includes antisymmetric components related to lattice rotations. To approximate the off-diagonal components, numerical integration techniques could be employed to approximate these components [158], provided that sufficient spatial resolution and boundary conditions are available.

Overall, the toolbox addresses a long-standing challenge in materials characterisation, linking high-resolution experimental measurements to actionable mechanical descriptors. It is particularly suited for applications in fatigue crack growth studies, microstructure-sensitive failure analysis, and defect quantification in advanced manufacturing processes. In addition, the toolbox aligns with the vision of Material Testing 2.0​ [159], emphasising the shift from conventional, idealised mechanical testing to automated, high-throughput, data-driven characterisation. The computerised processing pipeline allows for the efficient analysis of large datasets from advanced imaging techniques such as synchrotron X-ray tomography and high-resolution electron microscopy, thus supporting accelerated materials qualification and predictive maintenance workflows.

## Conclusions

We have formulated, implemented, and validated a general-purpose computational toolbox for extracting configurational forces and mixed-mode stress intensity factors from experimentally measured displacement or deformation gradient fields. The method implements path-independent energy integrals, including the *J*- and *M*-integrals, and introduces a novel mode decomposition approach that does not rely on predefined boundary conditions or external loads. Validation across synthetic and experimental datasets, including HR-EBSD, HR-TKD, and 3D-stereo DIC, demonstrates the toolbox’s robustness, accuracy, and versatility in 2D and 3D across different length-scales. The toolbox bridges a critical gap between experimental and theoretical mechanics frameworks by enabling quantitative, geometry-independent defect analysis.

## Supplementary Information

Below is the link to the electronic supplementary material.

Supplementary Material 1

## Data Availability

The data that support the findings of this study are available at https://github.com/Shi2oon/Defect_Descriptor.

## Footnotes

1 In the literature, typically, the *J*-integral is used to mean only the* J*_1_.

2 While energy release rate and strain energy release rate focus on energy dissipation during crack growth in linear elastic fracture mechanics (LEFM) according to Griffith’s theory [49,160]; potential energy release rate refers to the change in potential energy that drives this process. It is equivalent to the energy release rate and strain energy release rate, but framed from a system energy perspective and can be used for both LEFM and non-LEFM. We note that the term ‘rate’ is often misused in static contexts, as strictly it ought to be reserved for dynamic situations. Using ‘flux’ instead might be more appropriate in static conditions.

3 Global covers from having a finite number of defects to the formation of microcracks with random location and orientation.

4 The user can input the material anisotropic modulus, which can be used to calculate the equivalent Young’s modulus, shear modulus and Poisson’s ratio for cubic anisotropic materials based on ref. [90]. For non-cubic crystals, the user needs to input the equivalent elastic constants, which can be calculated using different methods, such as the Hashin-Shtrikman method [161] or the Elastic Self-Consistent (ELSC) model [162].

5 For more details about the Ramberg–Osgood relationship implementation, see the Supplementary information: B.

6 Within limits outlined in the Supplementary Information: A.

7 See Supplementary information: B for a case study on an elastoplastic material.

## Code availability

The MATLAB-based toolbox developed in this study is openly available under an MIT license. A permanently archived version of the code, including example datasets and documentation, is accessible via Zenodo at 10.5281/zenodo.6411484, ensuring compliance with the FAIR (Findable, Accessible, Interoperable, and Reusable) principles. A continuously maintained and updated toolbox version is hosted on GitHub at https://github.com/Shi2oon/Defect_Descriptor.
