# Supplementary material for: Bridging experiments and defects’ mechanics: a data-driven toolbox for configurational force analysis
Source: Eng Comput. 2026 Jan 14;42(1):21. doi: 10.1007/s00366-025-02262-5 (PMC12804342; doi:10.1007/s00366-025-02262-5)
Supplement: Supplementary file 1 — Supplementary Material 1 [file 366_2025_2262_MOESM1_ESM.docx]

Supplementary information of Bridging experiments and defects’ mechanics: A Data-driven toolbox for configurational force analysis

Abdalrhaman Koko ^a,b,^[[1]](#footnote-2)^^, Alya Abdelnour ^c^, Thorsten H. Becker ^d^, and T. James Marrow ^b^

^a^ National Physical Laboratory, Hampton Road, Teddington TW11 0LW, United Kingdom

^b^ Department of Materials, University of Oxford, Oxford OX1 3PH, United Kingdom

^c^ Department of Mechanical Engineering. University of Khartoum, Khartoum, Sudan

^d^ Centre for Materials Engineering, Department of Mechanical Engineering, University of Cape Town, Cape Town, South Africa

### Uncertainty due to noise and crack tip position

The key sources of errors in this analysis were assessed. First_,_ a normally distributed random noise was incrementally induced from zero to 10% on all the strain components. Figure 1a shows the induced error in each component, with K_II_ being highly influenced by noise. The dashed upper pound line indicates that this method is highly vulnerable to noise compared to a technique that uses total elastic displacement, e.g., a 6% noise induces a convergence error of 7.6 ± 18.8 %, 16.8 ± 35.5% and 4.2 ± 11.2 % in K_I_, K_II_ and K_III_, respectively, compared to 3.8%, 3% and 0.8% when using displacement field subjected to same noise [1,2]. This is because the induced noise on three displacement components will be diluted when the derivatives are calculated. In contrast, the induced noise on the nine strain components will directly affect the analysis. Calculating the strains next to the crack flanks is critical in mixed mode; this region cannot be masked or excluded, especially in mode I, but can be carefully extrapolated from the displacement gradients.


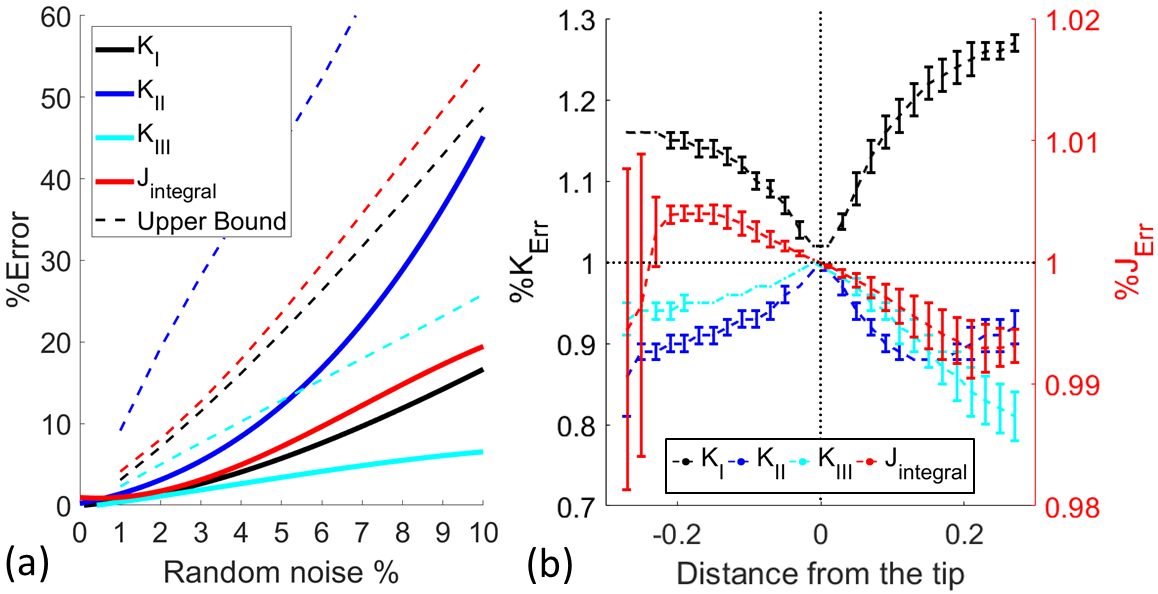


Figure 1: Error due to (a) noise and (b) crack tip accuracy.

Second, the sensitivity to crack tip location was assessed using the accurately crack tip positioned at the origin (0,0) coordinate. The error in accurately locating the crack increases if the location is assumed ahead relative to behind the crack (Figure 1b). This is due to the nature of the analytical field, as there is symmetry on the crack edges, but the magnitude at the crack tip gradually changes with distance. A detailed analysis of two-dimensional errors reveals a distinct behaviour for each component of SIFs and the sum of all components in the *J*-integral, which influences both magnitude and convergence.

### Additional case study: Curved crack in elastoplastic aluminium

To evaluate the robustness of the toolbox under conditions involving inelastic deformation, a simulated crack in a high-strength alloy was generated using a finite element model in Abaqus. A stationary, curved crack was embedded in a 2D plane-stress shell geometry (Figure 2a), and the deformation was driven to the post-yield regime by applying a nominal displacement to open on one side of the crack, resulting in strain energy release of 3160 J m^-2^ at the crack. The simulation used the Ramberg–Osgood model to capture the material’s non-linear elastic–plastic response, as described by equation (1).

| $E\varepsilon=\sigma+\alpha\sigma\left( \frac{\sigma}{\sigma_{0}} \right)^{n-1}$ | 1 |
| --- | --- |

where the hardening parameters were taken as: yield stress ($\sigma_{0}$) is 193 MPa, the yield offset ($\alpha$) = 1.24, exponent ($n$) = 26.67, Young's modulus ($E$) = 210 GPa, and Poisson ratio ($\nu$) of 0.3.


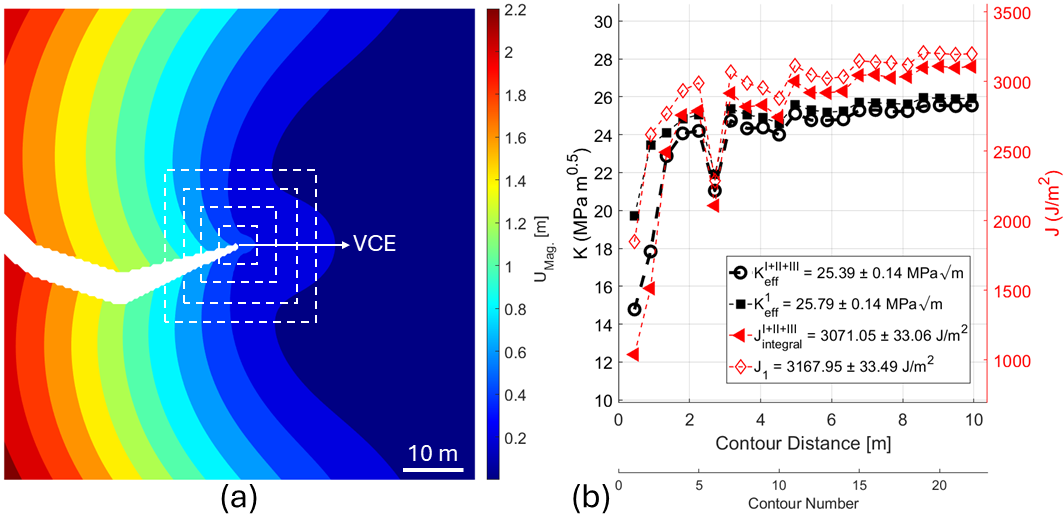


Figure 2: Analysis of a 2D displacement field for a curved crack in an elastoplastic material using the computational toolbox: (a) Simulated displacement magnitude field (U_mag_) for a curved crack under elastic–plastic deformation, computed in Abaqus and injected into the toolbox for fracture parameter extraction. The white dashed contours represent integration paths for the EDI method around the virtual crack extension (VCE) direction. (b) Convergence of the effective stress intensity factor ($K_{\mathrm{eff}}$) and *J*-integral with increasing contour number. The reference *J*-integral from Abaqus is 3660 J/m^2^.

The resulting displacement field from the simulation was exported from Abaqus and injected into the toolbox as an input. To account for stress contributions, the toolbox internally computes stress tensors from the strain field using the full elastic–plastic constitutive law by solving the inverse Ramberg–Osgood relation (equation 1) starting with an initial guess for the stress ($\sigma^{0}$).

| $\sigma^{0}=E\varepsilon$ | 2 |
| --- | --- |

Then, via the Newton-Raphson method, we can iteratively calculate stress ($\sigma$) from strain ($\varepsilon$) as outlined below, starting from calculating the deviatoric stress ($\sigma^{\mathrm{dev}}$):

| $\sigma^{\mathrm{dev}}=\sigma^{k}-\frac{1}{3}tr\left( \sigma^{k} \right) I$ | 3 |
| --- | --- |

where $k$ is the number of iterations (i.e., $k$=0, 1, 2, …, 1000) and $I$ is the identity matrix. This is followed by calculating the equivalent von Mises stress ($\sigma_{\mathrm{eq}}^{k}$):

| $\sigma_{\mathrm{eq}}^{k}=\sqrt{\frac{3}{2}\sigma^{\mathrm{dev}}:\sigma^{\mathrm{dev}}}$ | 4 |
| --- | --- |

We evaluate the vector-valued residual

| $f\left( \sigma^{k} \right)=\varepsilon-\frac{\sigma^{k}}{E}-\alpha\left( \frac{\sigma_{\mathrm{eq}}^{k}}{\sigma_{0}} \right)\frac{\sigma^{\mathrm{dev}}}{\sigma_{\mathrm{eq}}^{k}}$ | 5 |
| --- | --- |

Approximate the Jacobian to update the stress

| $\sigma^{k+1}=\sigma^{k}-\left( \frac{\partial f}{\partial\sigma} \right)^{-1}f$ | 6 |
| --- | --- |

Then check for convergence, i.e., the Frobenius norm, against the tolerance, which is set in the toolbox as 10^-6^

| $\left\Vert\sigma^{k+1}-\sigma^{k} \right\Vert_{\mathrm{Frobenius}}<tolerance$ | 7 |
| --- | --- |

Once convergence is achieved or the number of iterations is reached, the calculated stress fields are applied in the equivalent domain integral (EDI) formulation to estimate the mixed-mode stress intensity factors (SIFs) and the *J*-integral in the virtual crack extension (VCE) direction. Path independence was evaluated by inspecting convergence with an expanding domain.

As shown in Figure 2b, the computed *J*-integral (or $J_{1}$) converges to 3167.95 ± 33.49 J m^-2^, which is in excellent agreement with the reference value from Abaqus postprocessing (3160 J m^-2^), with an effective stress intensity factor ($K_{\mathrm{eff}}$) of 25.79 ± 0.14 MPa m^0.5^. Minor fluctuations in the contours arise due to local numerical noise near the crack tip. Also, noticeably, there is a slight difference between the *J*-integral calculated directly from the field and $J_{1}^{I+II+II}$ calculated from fields after decoupling of mode I–III due to the superimposed auxiliary field needed for decoupling. Nonetheless, the agreement between simulated and extracted *J*-integrals validates the toolbox’s ability to resolve nonlinear fracture fields, including contributions from both elastic and plastic deformation components.

Note that for elastoplastic materials, the *J*-integral becomes effectively path-independent if the integration domain fully encloses the plastic zone, ensuring all internal energy dissipation is captured. In this benchmark, the assumed quasi-static monotonic loading and the increasing contour size ensure that the plastic dissipation region is engulfed, leading to the observed convergence of the J-integral value.

### Influence of DVC’s out-of-plane displacement on mode III decomposition

A three-dimensional finite element model was developed in Abaqus to investigate mixed-mode crack behaviour under complex loading conditions. The geometry consists of a rectangular block containing a pre-existing straight crack front, oriented parallel to the global x-axis. The crack was modelled using hexahedral C3D8R (8-node linear brick, reduced integration) elements. The model assumes an isotropic, linear elastic material with Young’s modulus of 210 GPa and Poisson’s ratio of 0.3. To induce a non-trivial stress state at the crack tip, asymmetric boundary conditions were applied: tensile displacement was imposed on one face of the specimen, while compressive displacement was applied on the opposite end.

As shown in Figure 3a-c, this configuration promotes a combination of mode I (opening), mode II (sliding), and mode III (tearing) fracture mechanisms, thereby creating a mixed-mode loading scenario. The stress concentration and the asymmetric stress fields surrounding the crack tip confirm the presence of a strong mixed-mode condition. Notably, the stress contours exhibit a clear twist in the stress field (particularly in views Figure 3b and Figure 3c), which indicates significant mode III contribution in addition to modes I and II. To evaluate the fracture response, the SIFs were computed, assuming the crack propagation direction remains parallel to the x-axis.


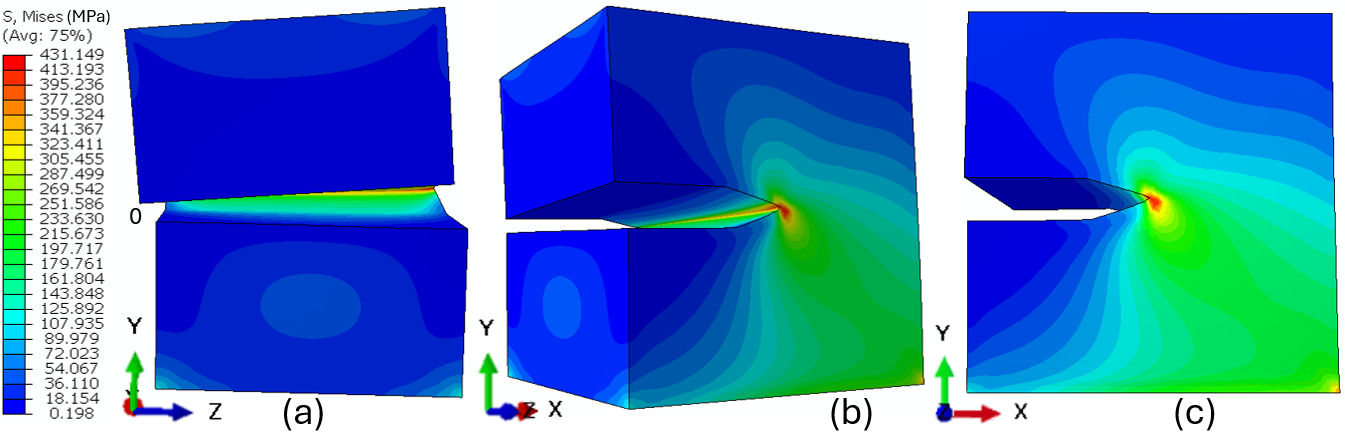


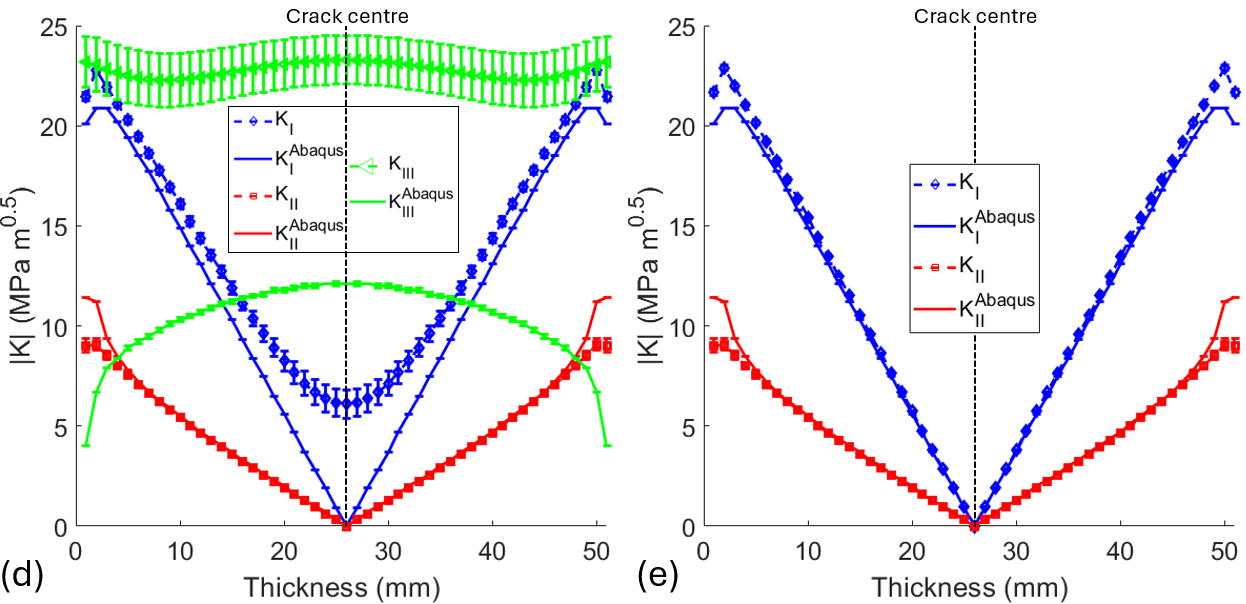


Figure 3: Von Mises stress distribution near a 3D straight crack front under mixed-mode loading: (a) YZ-plane view showing vertical stress concentration; (b) isometric view highlighting crack front twisting; (c) XY-plane view illustrating asymmetric stress field indicative of combined mode I, II, and III loading. Comparison of mode I, II, and III stress intensity factors across the crack front thickness: (d) SIFs computed using full 3D displacement field (including out-of-plane U_Z_​); (e) SIFs computed assuming U_Z_​ = 0. Symbols denote values from the toolbox; solid lines denote Abaqus *J*-integral-based results.

To validate the SIFs computed by the toolbox, the full 3D displacement field was extracted from the Abaqus model. The toolbox evaluates the SIFs by processing 2D slices of the displacement field in the X–Y plane along discrete positions in the Z direction (thickness), effectively reconstructing the mixed-mode fracture parameters across the crack front.

Figure 3d compares the SIFs obtained using the toolbox and those extracted from Abaqus (via the *J*-integral method) when the full 3D displacement field, including the out-of-plane component U_z_, is used. In contrast, Figure 3e shows the same comparison assuming U_z_ = 0, effectively reducing the problem to 2D plane strain. The results indicate strong agreement between both methods for mode I and mode II components, particularly near the crack centre. However, including the out-of-plane displacement is essential to capture the mode III contribution, which is otherwise absent in the 2D approximation.

The observed overestimation of the mode III SIF in Figure 3d can be attributed to a fundamental limitation in applying conventional mode decomposition techniques, originally designed for 2D or stereo-DIC displacement fields, to full 3D displacement data. The toolbox employed in this study reconstructs SIFs by decomposing the displacement field into individual mode components using near-tip asymptotic fields. While this approach performs reliably in 2D and stereo-DIC settings (where out-of-plane displacements are either absent or limited to a single transverse component), its direct extension to 3D fields introduces inconsistencies, particularly for mode III.

In 3D, ensuring equilibrium of the stress field, especially in the far field, becomes critical for accurate mode III characterisation. However, the standard decomposition approach does not inherently enforce the global equilibrium conditions required for accurate recovery of the anti-plane shear field. As a result, this leads to non-physical contributions to the calculated mode III field, particularly affecting the convergence of the $J_{1}^{III}$-integral and resulting in its overestimation. This discrepancy disappears in Figure 3e, where the out-of-plane displacement is artificially suppressed, effectively reducing the problem to 2D [3]. This simplification avoids the pitfalls associated with incomplete 3D equilibrium enforcement and yields SIFs in better agreement with Abaqus, although at the cost of neglecting real mode III contributions.

## References

[1] Molteno MR, Becker TH. Mode I-III Decomposition of the J -integral from DIC Displacement Data. Strain 2015;51:492–503. https://doi.org/10.1111/str.12166.

[2] Amiot F, Bornert M, Doumalin P, Dupré J ‐C., Fazzini M, Orteu J ‐J., et al. Assessment of Digital Image Correlation Measurement Accuracy in the Ultimate Error Regime: Main Results of a Collaborative Benchmark. Strain 2013;49:483–96. https://doi.org/10.1111/str.12054.

[3] Molteno MR. Measuring fracture properties using digital image and volume correlation: decomposing the J-integral for mixed-mode parameters. Doctor of Philosophy. Stellenbosch University, 2017.

1. Corresponding author. E-mail address: [abdo.koko@npl.co.uk](mailto:abdo.koko@npl.co.uk) [↑](#footnote-ref-2)
